# Storkhead box 2 and melanoma inhibitory activity promote oral squamous cell carcinoma progression

**DOI:** 10.18632/oncotarget.8495

**Published:** 2016-03-30

**Authors:** Tomonori Sasahira, Yukiko Nishiguchi, Rina Fujiwara, Miyako Kurihara, Tadaaki Kirita, Anja Katrin Bosserhoff, Hiroki Kuniyasu

**Affiliations:** ^1^ Department of Molecular Pathology, Nara Medical University, Kashihara, Japan; ^2^ Department of Oral and Maxillofacial Surgery, Nara Medical University, Kashihara, Japan; ^3^ Institute of Biochemistry, Friedrich-Alexander University of Erlangen-Nürnberg, Erlangen, Germany

**Keywords:** STOX2, MIA, metastasis, multidrug resistance, oral cancer

## Abstract

**Background:**

Storkhead box protein 2 (STOX2) is a transcriptional factor associated with pre-eclampsia with fetal growth restriction. We recently reported that melanoma inhibitory activity (MIA) promotes oral squamous cell carcinoma (OSCC) progression. However, the relationship between STOX2 and MIA remains unknown in malignancies.

**Methods:**

We used immunohistochemistry and PCR to investigate MIA and STOX2 expression in OSCC. We also performed functional analysis in human OSCC cells.

**Results:**

*MIA* and *STOX2* mRNA levels were higher in OSCCs than in normal oral epithelial cells, and upregulation of *STOX2* was significantly correlated with overexpression of *MIA*. Immunostaining for STOX2 was associated with nodal metastasis (P = 0.0002) and MIA expression (P < 0.0001). Furthermore, MIA expression (P = 0.0035) and STOX2 expression (P = 0.0061) were associated with poor outcome in OSCCs. *In vitro* analysis using OSCC cells revealed that *MIA* increased expression of STOX2 by paracrine manner. Moreover, STOX2 accelerated OSCC cell growth, invasion, suppressed apoptosis, and enhanced resistance to paclitaxel, cisplatin, and 5-FU.

**Conclusions:**

Our results suggest that MIA-STOX2 signaling may be a useful diagnostic and therapeutic target in OSCCs.

## INTRODUCTION

Head and neck cancer, including oral squamous cell carcinoma (OSCC), is the sixth most common cancer worldwide [1]. In the United States, OSCC is likely responsible for an estimated 8,650 deaths in 2015 [2], and the mortality rate of OSCC in Japan is 3.7 per 100,000 [3]. The overall 5-year survival rate of OSCC has remained at about 50% over the past 30 years [4]. Although paclitaxel, docetaxel, cisplatin, and 5-fluorouracil (5-FU) are regularly used to treat OSCC, multidrug resistance (MDR) of cancer cells has been reported [5–7]. To improve clinical outcomes, early diagnosis and treatment of OSCC will be critical.

Melanoma inhibitory activity (MIA) belongs to the *MIA* gene family together with the homologous genes *MIA*2 and *MIA3* [8–10]. *MIA3* encodes transport and Golgi organization protein 1 (TANGO) [10]. MIA is a secretory protein and has been implicated in the progression of malignant melanoma [11, 12]. MIA has been reported to promote cell separation, migration, invasion, metastasis, and inhibit cancer cell apoptosis [11–14]. MIA is capable of binding cell surface integrin α_4_β_1_ and α_5_β_1_, indicating that MIA may act as a ligand for some integrins [15]. We also reported that MIA expression is upregulated by binding of high-mobility group box 1 (HMGB1) and nuclear factor kappa B (NFkB) p65 to the *MIA* promoter region. Upregulation of MIA accelerated OSCC progression, nodal metastasis, angiogenesis, and lymphangiogenesis by activation of the vascular endothelial growth factor (VEGF) [16, 17]. Furthermore, MIA expression is observed in gastric cancer, pancreatic cancer, breast cancer, glioma, and chondrosarcoma [8, 18–21]. However, little is known about downstream signaling partners of MIA in malignancies.

Comparison of transcriptional profiles using a cDNA microarray demonstrated that storkhead box protein 2 (STOX2) is downregulated in OSCC cells with *MIA* konocdown (unpublished data). STOX2 is an important paralogue of STOX1, a winged-helix domain-containing transcription factor that participates in trophoblast differentiation [22]. Transcriptional profiling reported aberrant expression of *STOX2* in the neural crest stem cells and pulmonary cells of the offspring of pregnant mouse models of asthmatic inflammation [23, 24]. Moreover, STOX2 expression was reduced in the decidual tissue of patients with fetal growth restriction (FGR) [25]. However, the role of STOX2 in tumors remains unknown. In the present study, we investigated the role of STOX2 in OSCC.

## RESULTS

### Expression of MIA and STOX2 in oral squamous cell carcinoma specimens

**Figure 1:**
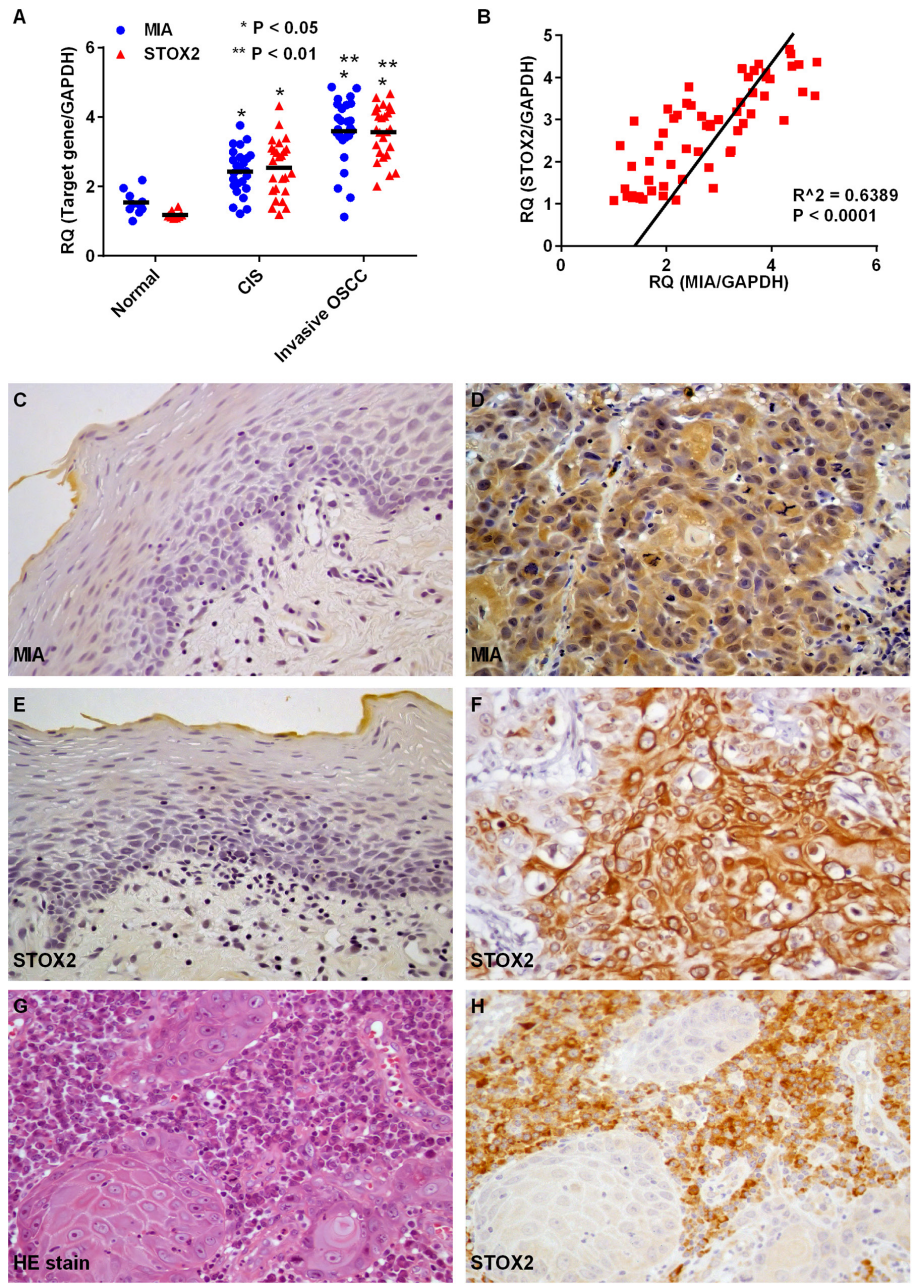
Expression of MIA and STOX2 in human OSCC cases **A.** Gene expression analysis using cDNA obtained by laser capture microdissection (LCM) in normal epithelium of the oral cavity and oral squamus cell carcinoma (OSCC). Levels of *MIA* and *STOX2* expression in carcinoma *in situ* (CIS) (P < 0.05) and invasive OSCC (P < 0.01) cases were high compared with that in the normal oral mucosa. Further, *MIA* and *STOX2* expression in patients with invasive OSCCs were higher than in patients with CIS (P < 0.05). **B.** Overexpression of *STOX2* was significantly associated with upregulation of *MIA* in OSCCs (P < 0.0001). **C–H.** Immunostaining of MIA and STOX2 in human OSCC cases. Weak and/or no expression of MIA (C) and STOX2 (E) were found in tumor free oral mucosa. Expression of MIA (D) and STOX2 (F) were observed in OSCCs. (G) Stromal plasma cells surrounding the OSCC cell nest. (H) Immunostaining of STOX2 was found in stromal plasma cells. Original magnification was 400×. HE; hematoxylin and eosin

We used qRT-PCR to assess expression of *MIA* and *STOX2* in OSCC (Figure 1A). To avoid the influence of stromal cell *MIA* and *STOX2* expression, microdissected-samples of normal oral epithelium, CIS, and invasive OSCCs were used. Expression of *MIA* and *STOX2* was higher in CIS and OSCC samples than in the normal oral mucosa (both, P < 0.01). Expression of *MIA* and was also higher in invasive OSCC than CIS samples (both, P < 0.01). Further, *STOX2* expression in tumors was significantly associated with *MIA* expression (P < 0.0001, Figure 1B). Next, we used immunohistochemistry to investigate MIA and STOX2 expression in 202 cases of OSCC (Figure 1C–1H). Little to no expression of MIA and STOX2 was detected in normal oral mucosa (Figure 1C and 1E), whereas membranous and/or cytoplasmic MIA and STOX2 was detected in OSCC cells (Figure 1D and 1F). We also confirmed that STOX2 is expressed in stromal plasma cells (Figure 1G and 1H). However, co-localization of STOX2 and MIA was hardly observed in OSCC samples by conventional immunohistochemistry and double immunofluorescent staining (data not shown). Immunostaining of MIA and STOX2 was observed in 42.1% (85/202) and 28.7% (58/202) of patients with OSCC, respectively. The relationship between MIA or STOX2 overexpression and clinicopathological characteristics is described in Table 1. Overexpression of MIA was only associated with nodal metastasis (P < 0.0001); this is consistent with the results of our prior report [16]. Immunoreactivity for STOX2 was observed in 44.7% (34/76) of the nodal metastasis-positive cases but only 19.1% (24/126) of the cases without nodal metastasis (P = 0.0002). Elevated expression of STOX2 was also correlated with overexpression of MIA in OSCCs (P < 0.0001). Of the 85 samples that stained positive for MIA, 43 (50.6%) also expressed STOX2, while of the 117 samples that contained no MIA, only 15 (12.8%) also expressed STOX2. STOX2 was observed in the cell membrane of plasma cells surrounding the OSCC cell nest (Figure 1G and 1H); however, its expression in plasma cells was not significantly associated with clinicopathological characteristics (data not shown).

**Table 1:** Relationship between MIA or STOX2 expression and clinicopathological parameters in OSCCs

Parameters	MIA		STOX2	
	negative	positive	negative	positive
Gender				
Male	80	55	93	42
Female	37	30	51	16
*P* value	0.6504		0.3242	
Age				
<-65	46	36	57	25
>65	71	49	87	33
*P* value	0.6666		0.7517	
Site				
Tongue	55	41	68	28
Gingiva	38	32	49	21
Other	24	12	27	9
*P* value	0.3713		0.8569	
Histological differentiation*				
Well	54	45	63	36
Moderately	50	34	66	18
Poorly	13	6	15	4
*P* value	0.4939		0.0622	
T classification				
Tis, T1	14	11	14	11
T2	29	22	38	13
T3	44	19	44	19
T4	30	33	48	15
*P* value	0.0906		0.2705	
Clinical stage				
I	14	11	14	11
II	29	16	37	8
III	41	20	43	18
IV	33	38	50	21
*P* value	0.0786		0.1370	
Nodal metastasis				
Negative	92	34	102	24
Positive	25	51	42	34
*P* value	< 0.0001		0.0002	
MIA				
Negative	-	-	102	15
Positive	-	-	42	43
*P* value	-		<0.0001	

Relationship between STOX2 expression and each parameters were calculated by χ^2^ test or Fisher's exact test. T classification and clinical stage were classified according to the TNM classification.

### Association between STOX2 expression and oral squamous cell carcinoma prognosis

**Figure 2:**
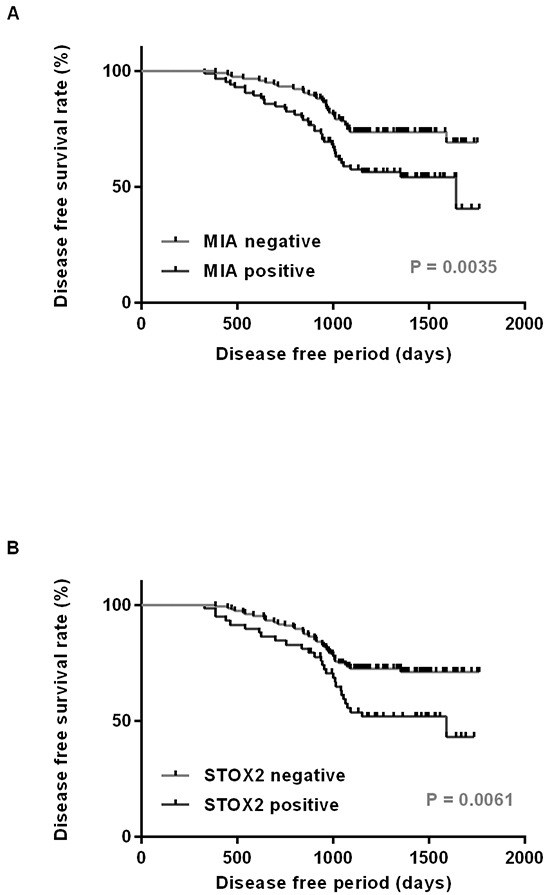
Disease free survival curve in OSCC cases Disease free survival was calculated by Kaplan–Meier method. The cases with expression of MIA **A.** and STOX2 **B.** had significantly worse prognosis than did those with tumors negative for these expressions (P = 0.0035 and P = 0.0061, respectively).

Local and nodal recurrence occurred in 68 of the 202 cases. Next, we performed a survival analysis using the Kaplan–Meier method. We found that disease-free survival of MIA-positive patients was significantly shorter than that of MIA-negative patients (P = 0.0035; Figure 2A). Moreover, STOX2 expression was associated with a poor OSCC outcome (P = 0.0061; Figure 2B). Next, we performed univariate and multivariate Cox proportional hazards analyses. Univariate analysis indicated that T factor (P = 0.0023), clinical stage (P = 0.0115), nodal metastasis (P = 0.0098), MIA expression (P = 0.0067), and STOX2 expression (P = 0.0088) were associated with poor OSCC outcome. In a multivariate analysis, MIA (P = 0.0114) and STOX2 expression (P = 0.0449) were independent predictors of disease-free survival in patients with OSCC (Table 2).

**Table 2:** Univariate and multivariate analysis of disease free survival

Parameters	Univariate analysis			Multivariate analysis		
	HR	95% CI	*P* value	HR	95% CI	*P* value
Gender						
M	1.00					
F	1.2081	0.7318–1.9571	0.4526			
Age						
≤65	1.00					
>65	0.8406	0.5215–1.3705	0.4812			
Site						
Tongue	1.00					
Other	1.1741	0.7290–1.9081	0.5100			
Histology						
Well	1.00					
Mod, Por	0.8258	0.5105–1.3300	0.4307			
T factor						
Tis-3	1.00			1.00		
T4	1.9362	1.2021–3.1397	0.0067	1.4427	0.8294–2.5103	0.1938
Clinical stage						
I–III	1.00					
IV	1.5797	0.9738–2.5453	0.0638			
Nodal metastasis						
Negative	1.00			1.00		
Positive	1.8821	1.1667–3.0363	0.0098	1.1509	0.6142–2.1261	0.6576
MIA						
Negative	1.00			1.00		
Positive	2.1513	1.3219–3.4696	0.0023	6.7357	1.4118–120.716	0.0114
STOX2						
Negative	1.00			1.00		
Positive	1.9418	1.1868–3.1336	0.0088	1.8507	1.0457–3.2264	0.0449

Univariate and multivariate analysis was calculated by means of Cox proportional hazard model. HR and 95% CI mean hazard ratio and 95% confidence intervals, respectively.

### Regulation of STOX2 expression by MIA in oral squamous cell carcinoma cells

**Figure 3:**
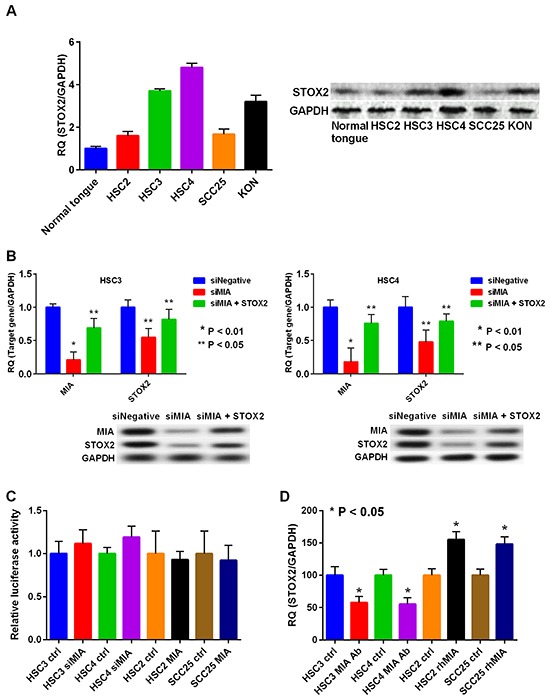
Expression and regulation of STOX2 in OSCC cells **A.** Expression levels of STOX2 determined by realtime RT-PCR (left) and immunoblotting (right) in the OSCC cells. The GAPDH expression levels were used as internal controls for STOX2. **B.** Gene (left) and protein (right) expression levels of MIA and STOX2 by treatment with *MIA* knockdown in HSC3 cells (top) and HSC4 cells (bottom). **C.** *STOX2* promoter activity was tested by luciferase reporter assay. **D.** Gene expression levels of *STOX2* by anti-MIA antibody or rhMIA treatment in OSCC cells. bar, standard deviation (SD). RQ; relative quantification.

To validate that OSCC cell lines also exhibited the characteristics observed in clinical samples, we evaluated STOX2 expression in cultured OSCC cells. Expression of STOX2 was higher in OSCC cells than in normal tongue mucosa cells (Figure 3A). Particularly, HSC3, HSC4, and KON cells, which possess invasive and/or metastatic ability, expressed higher levels of STOX2 than did HSC2 and SCC25 cells. Previously, we reported that HSC3 and HSC4 cells overexpress MIA [16, 17]. As we observed that expression of STOX2 correlated with MIA overexpression in OSCCs, we sought to investigate the possibility that MIA upregulates STOX2. We used siRNA to knockdown *MIA* expression in HSC3 and HSC4 cells and observed resultant reduction in STOX2 expression (Figure 3B). Furthermore, STOX2 expression levels were restored by treatment with *MIA* siRNA and *STOX2* transfection (Figure 3B). Although *MIA* did not significantly change in *STOX2* promotor activity (Figure 3C), STOX2 expression levels were increased or decreased under recombinant protein or antibody treatment for MIA in OSCC cells (Figure 3D). These results suggested that MIA increases the STOX2 expression levels by paracrine manner in OSCC cells.

### Function of STOX2 in oral squamous cell carcinoma cells

**Figure 4:**
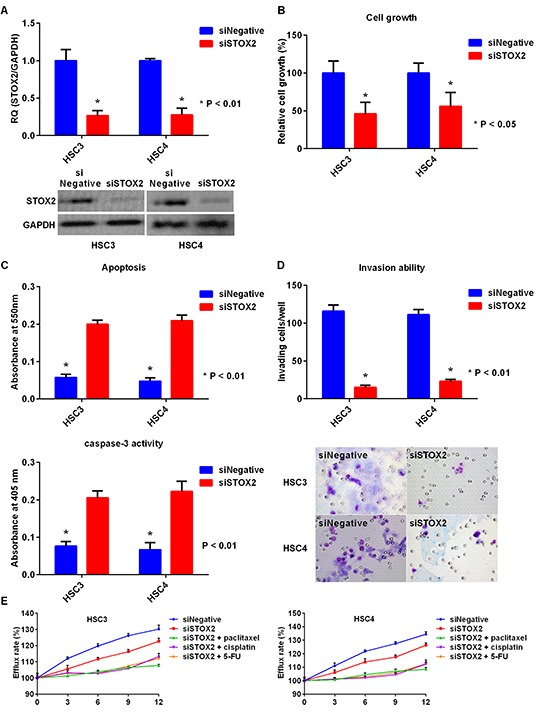
Effect of *STOX2* knockdown treatment in OSCC cells **A.** Gene (top) and protein (bottom) expression of STOX2 in OSCC cells by inhibition of *STOX2* mRNA expression. **B.** Effects of *STOX2* short interfering RNA (siRNA) treatment on cell growth in OSCC cells. **C.** The impact of *STOX2* siRNA on apoptosis (top) and activation of caspase-3 (bottom) in OSCC cells. **D.** Changes in invasive ability treated with *STOX2* knockdown in OSCC cells. **E.** Influence of anticancer drug resistance by treatment with *STOX2* siRNA and co-treatment with *STOX2* siRNA and paclitaxel, cisplatin, or 5-FU in HSC3 cells (left) and HSC4 cells (right). Error bar, standard deviation (SD). RQ; relative quantification.

To further examine the effects of STOX2 in OSCC cells, we used siRNA to knockdown *STOX2* expression in HSC3 and HSC4 cells (Figure 4A) and observed resultant reduction in cell growth (Figure 4B). In addition, apoptosis and caspase-3 activities were increased in *STOX2-*knockdown cells (Figure 4C). Furthermore, knockdown of *STOX2* decreased the ability of HSC3 and HSC4 cells to invade in a Boyden Chamber model (Figure 4D). Next, we analyzed the effect of STOX2 on OSCC cell tolerance to chemotherapeutic drugs using the MDR Assay Kit. This kit allows measurement of efflux of a fluorescent dye that binds to cell surface ABC transporters. Exposure to *STOX2* siRNA restored OSCC cell resistance to the anticancer drugs. Moreover, co-treatment with *STOX2* siRNA and paclitaxel, cisplatin, or 5-FU decreased MDR in HSC3 and HSC4 cells (Figure 4E).

**Figure 5:**
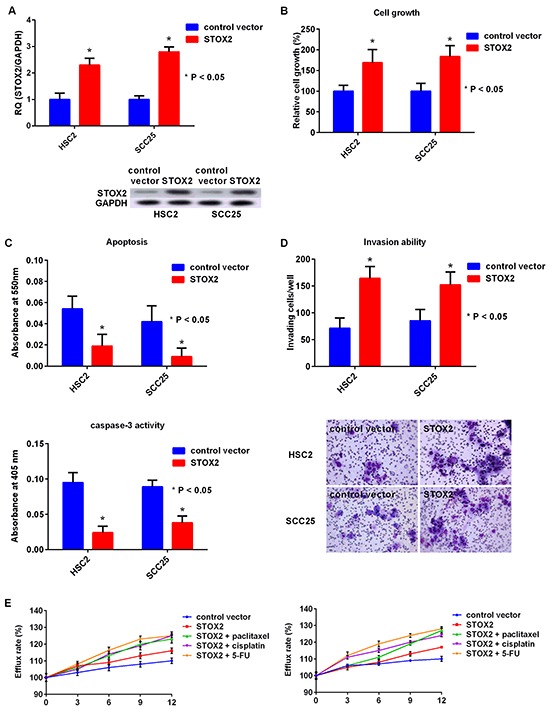
Effect of upregulation of *STOX2* in OSCC cells **A.** Gene (top) and protein (bottom) expression of STOX2 in OSCC cells by overexpression of *STOX2*. **B–E.** Effects of *STOX2* upregulation treatment on cell growth (B), apoptosis and activation of caspase-3 (C), invasive ability (D), and anticancer drug resistance (E) in OSCC cells. Error bar, standard deviation (SD). RQ; relative quantification.

Finally, we performed *STOX2* knockdown or upregulation treatment (Figure 5A). The growth of the HSC2 and SCC25 cells treated with STOX2 overexpression was restored compared with that of the cells treated with the control vector (Figure 5B). Moreover, apoptosis and caspase-3 activities were decreased in cells with *STOX2* upregulation (Figure 5C). The number of invading OSCC cells upon *STOX2* overexpression was significantly higher than that of cells with control treatment (Figure 5D). Further, MDR was increased in OSCC cells with *STOX2* overexpression (Figure 5E). These results suggested that STOX2 may elicit oncogenic functions in OSCC cells.

## DISCUSSION

Members of the *MIA* gene family appear to perform several tumor-related functions. MIA reportedly enhances progression and metastasis of malignant melanoma, chondrosarcoma, glioma, gastric, pancreatic, and breast cancer, as well as OSCCs [8, 11-21, 26, 27]. Additionally, we previously reported that the MIA2-integrin α_4_β_1_ pathway promotes invasion and dysregulation of the host immune system, suppressing OSCC apoptosis [28]. We also verified that TANGO promotes cell growth, invasion, angiogenesis, and lymphangiogenesis and confers resistance to apoptosis in OSCC [10]. Recent reports have revealed that MIA expression is induced by binding of SRY (sex-determining region Y)-box 10 (SOX10) to the *MIA* promoter region in malignant melanoma [26]. In OSCC, MIA is upregulated by intracellular HMGB1 and NFkB p65 and promotes tumor progression and nodal metastasis by inducing VEGF family-mediated upregulation of angiogenesis and lymphangiogenesis [16, 17]. Further, MIA possesses *p54^nrb^* promoter activity by activating transcription factor Y box-binding protein 1 (YBX1) [29, 30]. MIA is a ligand for the cell surface receptors integrin α_4_β_1_ and α_5_β_1_ and also binds with fibronectin to inhibit cell-to-stromal adhesion [13–15]. However, the role of MIA in OSCC is still unclear.

Unraveling the downstream signaling pathways of MIA in cancer cells may inform development of efficacious anti-cancer strategies. Thus, we here investigated expression of MIA and the potential downstream partner STOX2 in OSCC specimens. Immunoreactivity for MIA was correlated with nodal metastasis and poor prognosis of OSCC, findings consistent with our previous report [16, 17]. Moreover, we investigated whether MIA regulates STOX2 expression.

Expression of STOX2 was previously reported to be downregulated in term decidua cases with both pre-eclampsia and FGR [25]. However, the role of STOX2 in malignancies is not known. We observed that STOX2 expression is upregulated by MIA and promotes OSCC cell growth, invasion, resistance to anticancer drugs, and inhibits apoptosis. STOX2 overexpression was also correlated with nodal metastasis, poor prognosis, and expression of MIA in OSCC. Smith *et al.* previously reported that in cDNA microarray analysis *STOX2* expression is associated with prognosis in colon cancer, and our results are largely consistent with their findings [31]. Conversely, CpG island hypermethylation of *STOX2* promoter region reportedly reduces STOX2 expression in colon adenoma and adenocarcinoma [32, 33]. However, methylation of *STOX2* was not observed in OSCC cells (data not shown). In this study, MIA was increased the expression of STOX2 by paracrine manner. However, direct binding of *MIA* to *STOX2* promoter regions was not observed in OSCC cells. Nevertheless, it remains unknown whether STOX2 is regulated by DNA methylation and other members of the *MIA* gene family. Further *in vitro/in vivo* analysis and large-scale clinicopathological investigations using OSCC and non-tumor oral mucosa will be required to define the methylation and promoter activity of *STOX2*.

Cancer stroma is essential for cancer cell invasion, metastasis, and anticancer therapy [34]. Although STOX2 expression was observed in the stromal plasma cells surrounding OSCC, its significance remains unclear. Although most stromal cells can inhibit tumor cells, the stroma is altered by malignant transformation and may eventually promote growth, invasion, and metastasis of cancer cells [34]. Recently, Fujimoto *et al.* reported that immunoglobulin G4 (IgG4)-positive plasma cell infiltration in the stroma could contribute to favorable outcomes in lung SCC and adenocarcinoma [35]. Although cancer stromal STOX2 may improve host anticancer immunity, further studies will be required to validate the role of STOX2 in tumor stroma.

Paclitaxel, docetaxel, cisplatin, and 5-FU are usually used to treat OSCC [6]. However, MDR remains an important problem in cancer therapy. Molecular-targeted therapy with cetuximab, an anti-epidermal growth factor receptor-specific chimeric monoclonal antibody, has been also demonstrated to inhibit tumor growth, cancer cell invasion, angiogenesis, and metastasis, thereby improving overall survival in head and neck OSCC patients [36, 37]. However, the therapeutic efficacy of cetuximab in patients with OSCC remains controversial. Classical MDR is characterized by upregulation of ABC transporter genes, such as *ABCB1* and *ABCG2,* that transport anticancer agents out of the cell and confer tumor cell resistance to those drugs [5, 7, 38]. In this research, we confirmed that STOX2 accelerates acquisition of MDR in OSCC. Further, co-treatment with *STOX2* knockdown and paclitaxel, cisplatin, or 5-FU decreased MDR. However, combination therapy with anticancer drugs is the most commonly applied therapeutic strategy for OSCC, and many other genes may be involved OSCC MDR. In addition, the relationship between activation of STOX2 and other anticancer treatments, including radiation therapy, heavy ion radiotherapy, and hyperthermia, need to be investigated. Appropriate animal and *in vitro* models will be required for further investigations.

In conclusion, we determined that STOX2 promotes cell growth, inhibits apoptosis, confers MDR, and contributes to nodal metastasis and poor survival. We also showed that MIA facilitates STOX2 expression. To our knowledge, these results are novel findings, and we here present the first detailed report on the oncogenic functions of STOX2. Our findings indicate that STOX2 may be a useful diagnostic and therapeutic target in OSCC. Further investigations into STOX2 may provide new insights into molecular tumor markers of OSCC. This may eventually improve the quality of life and overall survival of patients with the disease.

## MATERIALS AND METHODS

### Surgical specimens

Specimens collected from 202 patients with primary OSCC (135 men and 67 women; age range: 42–85 years; mean age: 67.8 years) were formalin-fixed and paraffin-embedded. We also analyzed expression of *MIA* and *STOX2* in 10 frozen samples of non-tumor oral mucosa (6 men and 4 women; age rage, 29–46 years; mean: 36.8 years) and 25 samples of carcinoma *in situ* (CIS) (15 men and 10 women; age range, 58–68 years; mean: 64.2 years) and invasive OSCC (18 men and 7 women; age range, 55–72 years; mean: 65.6 years). The study was performed according to the Declaration of Helsinki, and was approved by the Medical Ethics Committee of Nara Medical University, Kashihara, Japan. All specimens were obtained from randomly selected patients at Nara Medical University Hospital, Kashihara, Japan, without preoperative therapy. Tumor staging was assessed according to the Union for International Cancer Control TNM classification system (seventh edition), and OSCC histological grade was classified according to the World Health Organization criteria. Medical records and prognostic follow-up data were obtained from the hospital's patient database. The follow-up period ranged from 329 to 1,764 days (mean: 1,179 days)

### Immunohistochemistry

Consecutive 3-mm sections were cut from each specimen block. Immunohistochemical analysis was performed using the EnVision + Dual Link System (Dako, Carpinteria, CA, USA). Antigens were retrieved by microwaving in citrate buffer at 95°C for 45 min. After endogenous peroxidase blocking with 3% H_2_O_2_-methanol, anti-MIA antibody (R&D Systems, Minneapolis, MN, USA, clone 294203) specimen were stained with anti-STOX2 antibody (Santa Cruz Biotechnology, Santa Cruz, USA, clone T-20) diluted at 0.5 mg/ml, color-developed with diaminobenzidine (DAB) solution (Dako), and counterstained with Meyer's hematoxylin (Sigma-Aldrich, St, Louis, MO, USA). We categorized immunoreactivity into four grades based on AS [39]: Grade 0, AS of 0; Grade 1, AS of 2–4; Grade 2, AS of 5 or 6; and Grade 3, AS of 7 or 8. Patients with grades 2 and 3 immunoreactivity were considered immunologically positive, as previously established [28]. The slides were examined by a pathologist (TS) blinded to the clinicopathological data.

### Laser capture microdissection

Laser capture microdissection (LCM) was performed to selectively extract total RNA from OSCC specimens. Tissue sections (7-mm) were prepared from each paraffin block and stained with hematoxylin and eosin. Slides were transferred for microdissection using a Pix Cell II laser capture microscope (Arcturus, Mountain View, CA, USA) according to the manufacturer's instructions. Approximately 5,000 tumor cells were microdissected from each sample. Total RNA was extracted using TRIzol reagent (Invitrogen, Carlsbad, CA, USA).

### Cell culture

The human OSCC cell lines HSC2, HSC3, HSC4, and KON were obtained from the Japanese Collection of Research Bioresources (JCRB) Cell Bank, Osaka, Japan, and SCC25 cells were purchased from the American Type Culture Collection (ATCC), Manassas, VA, USA. All cell lines were authenticated by JCRB and ATCC using short tandem repeat (STR) analysis. Total RNA from the normal tongue was purchased from Biochain (Newark, CA, USA) and used as a control. Cells were maintained in Dulbecco's modified Eagle's medium (DMEM; Wako Pure Chemical, Osaka, Japan) supplemented with 10% fetal bovine serum (Nichirei Biosciences, Tokyo, Japan) in 5% CO_2_ in air at 37°C. Anti-MIA antibody (R&D Systems) was used for neutralizing MIA in cultured medium at 2 μL/mL concentration. Further, 20 mM of recombinant human MIA (rhMIA) (Abnova, Taipei, Taiwan) treatment was performed. Moreover, cells were treated with 10 nM paclitaxel (Wako Pure Chemical), cisplatin (Wako Pure Chemical), and 5-FU (Wako Pure Chemical).

### RNA extraction and quantitative reverse-transcription polymerase chain reaction

Total RNA was extracted using a TRIzol reagent (Invitrogen), and total RNA (1 mg) was synthesized using a ReverTra Ace qPCR RT Kit (Toyobo, Osaka, Japan). Quantitative reverse-transcription polymerase chain reaction (qRT-PCR) was performed on a StepOnePlus Real-Time PCR System (Applied Biosystems, Foster City, CA, USA) using TaqMan Fast Universal PCR Master Mix (Applied Biosystems) and analyzed using the relative standard curve quantification method. The PCR conditions used were selected according to the manufacturer's instructions, and glyceraldehyde-3-phosphate dehydrogenase (*GAPDH*) mRNA was amplified as an internal control. TaqMan Gene Expression Assays of *STOX2* (identification number: Hs01391761_m1), *MIA* (identification number: Hs00197954_m1), and *GAPDH* (identification number: Hs03929097_g1) were purchased from Applied Biosystems. All PCRs were performed in triplicate.

### Immunoblotting

Whole-cell lysate was obtained using M-PER mammalian protein extraction reagent (Thermo Fisher Scientific, Rockford, IL, USA) according to the manufacturer's protocol, and 50 mg of the lysate was subjected to immunoblotting in 12.5% SDS-PAGE, followed by electrotransfer to polyvinylidene fluoride (PVDF) membranes (Thermo Fisher Scientific). The filters were incubated with MIA (R&D Systems, clone 294203) and STOX2-directed antibodies (Santa Cruz Biotechnology, clone T-20) and then with peroxidase-conjugated IgG (MBL, Nagoya, Japan). The immune complex was visualized by ECL Western blotting detection system (GE Healthcare, Amersham place, UK). Anti-GAPDH antibody (Santa Cruz Biotechnology, clone V-18) was used as an internal control.

### Transient transfection

To inhibit endogenous gene expression, cells were treated with short interfering RNA (siRNA). Silencer Select RNAi for *MIA* (identification number: s228273) and *STOX2* (identification number: s32515) were purchased from Ambion (Austin, TX, USA). AllStars Negative Control siRNA (Qiagen) was used as a control. Further, *STOX2* cDNA was amplified by PCR and sub-cloned into pcDNA3.1 (Invitrogen). Twenty nanomoles of siRNA and *STOX2*-pcDNA3.1 were transfected with Lipofectamine 2000 (Invitrogen) according to the manufacturer's recommendations.

### Cell growth, apoptosis, and anticancer resistance assays

Cells were seeded at density of 2,000 cells per well in 96-well tissue culture plates and incubated for 48 h at 37°C. Cell growth was assessed using a Cell Counting Kit-8 (Dojindo Laboratories, Kumamoto, Japan), and apoptotic cells were detected using the APOPercentage Apoptosis assay (Biocolor, Carrickfergus, County Antrim, UK). The MDR was monitored using MarkerGene Multiple Drug Resistance Microtiterplate Assay Kit (Marker Gene Technologies, Eugene, OR, USA). Absorbance at 405 nm (for the measurement of caspase-3 activity), 450 nm (for the measurement of cell growth), and 550 nm (for the measurement of apoptosis) were measured using a Multiskan GO Microplate Spectrophotometer (Thermo Fischer Scientific). We also confirmed activation of caspase-3 using CaspACE Assay system, Colorimetric (Promega, Madison, Wisconsin, USA), according to the manufacturer's protocol. Furthermore, MDR was measured in a SpectraMax M2 multi detection microplate reader (Molecular Devices, Sunnyvale, CA, USA) at Em 504 nm and Ex 538 nm. All experiments were performed in triplicate.

### Cell invasion assay

A modified Boyden chamber assay was performed using BD BioCoat cell culture inserts coated with Type IV Collagen (BD Biosciences, Bedford, MA, USA), as previously described. Briefly, cells were suspended in 500 ml of DMEM and placed in the insert. After incubation for 48 h at 37°C, the filters were dyed with a Diff-Quick staining kit (Siemens Healthcare Diagnostics, Newark, DE, USA), and the stained cells were counted in whole inserts at 100× magnification. Each experiment was repeated at least three times.

### Luciferase reporter assay

The LightSwitch promoter reporter GoClone collection for *STOX2* and negative promoter vector were purchased from Active Motif (Carlsbad, CA, USA). Subsequently, cells were transfected with the reporter plasmid and negative siRNA, *MIA* siRNA, control vector, or *STOX2*-pcDNA3.1 using Lipofectamine 2000 (Invitrogen). All experiments were performed in triplicate. After 24 h, cells were harvested and luciferase activities were tested using the Dual-Luciferase Reporter System (Promega Corporation) according to the manufacturer's instructions.

### Statistical analysis

Statistical analyses were performed using the χ^2^ test, Fisher's exact test, one-way analysis of variance, Student's *t*-test, Welch's *t*-test, and simple regression analysis. Disease-free survival was analyzed using the Kaplan–Meier method, and the groups were compared using the log-rank test. Univariate and multivariate analyses were performed using the Cox proportional hazards model. All statistical analyses were conducted using JMP8 (SAS Institute, Cary, NC, USA).
